# Rutoside and Ascorbic Acid in the Treatment of Schamberg Pigmented Purpuric Dermatosis

**DOI:** 10.7759/cureus.14592

**Published:** 2021-04-20

**Authors:** Alexandra J Morquette, Jason B Lee, Shoshana K Grossman, Sylvia Hsu

**Affiliations:** 1 Dermatology, Temple University Lewis Katz School of Medicine, Philadelphia, USA; 2 Dermatology and Cutaneous Biology, Thomas Jefferson University, Philadelphia, USA

**Keywords:** schamberg disease, rutoside, pigmented purpuric dermatosis, ascorbic acid, flavonoid, vitamin c

## Abstract

Schamberg disease is a type of pigmented purpuric dermatosis (PPD), which is a benign cutaneous capillaritis characterized by macules and patches most commonly found on the lower extremities. Rutoside and ascorbic acid have been shown in previous cases to be efficacious in the treatment of PPD lesions due to their free radical scavenging effect on capillaries. We present the case of a 19-year-old woman with Schamberg disease who achieved complete clearance of lesions within four months of daily rutoside and ascorbic acid treatment. The goal of this case report is to further demonstrate the effectiveness of this treatment and to call for investigation of its use as a standard of care in PPD.

## Introduction

Schamberg disease, the most common type of pigmented purpuric dermatosis (PPD), is a capillaritis characterized by reddish-brown macules and patches, most commonly distributed on the lower legs, but may appear on other parts of the body [1-3]. PPD usually is not associated with any symptoms, but pruritus may occur with some lesions. Although not completely understood, theorized etiologies of PPD include drug or food additive reaction, viral infection, or exercise. Lesions can wax and wane, persist with time, or resolve over months to years. The current standard of care for PPD includes topical steroids for those with pruritus, avoiding or discontinuing triggers, and the use of compression stockings. Recent studies have shown that the use of rutoside and ascorbic acid has been effective in the treatment of PPD [4,5]. In this case report, we present a patient with PPD who experienced marked improvement with oral rutoside and ascorbic acid therapy.

## Case presentation

**Figure 1:**
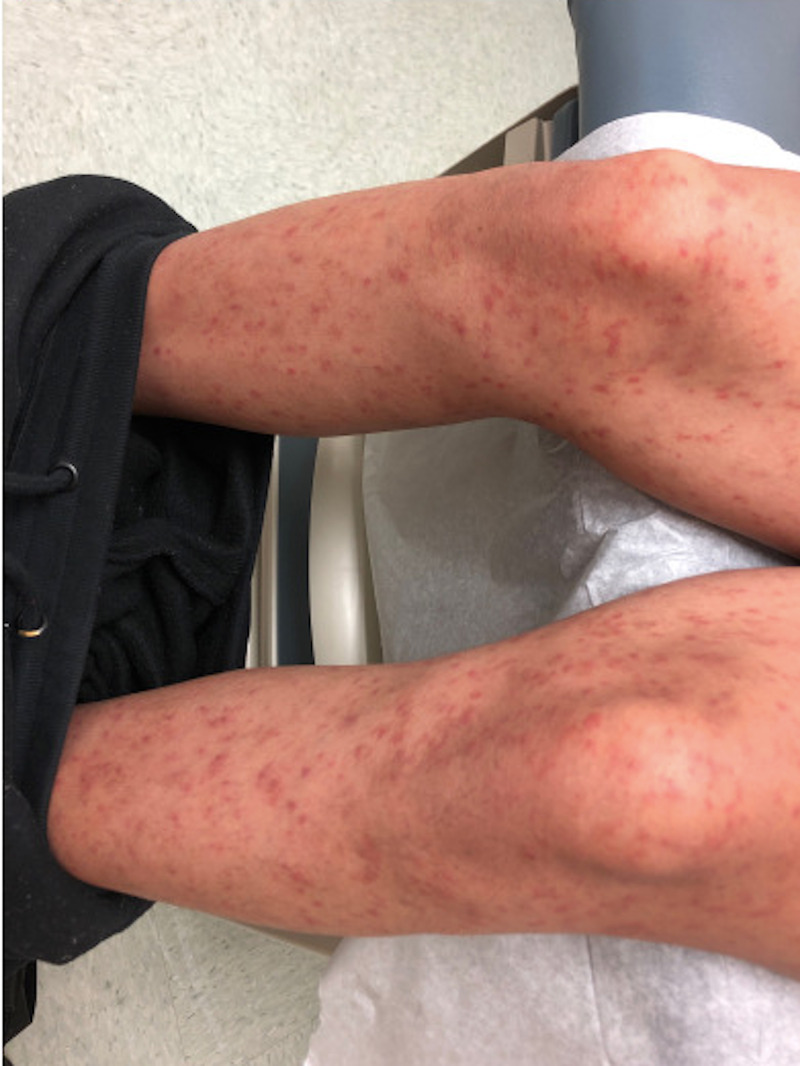
Schamberg disease. Non-blanching erythematous and brown papules and macules on the lower extremities.

**Figure 2:**
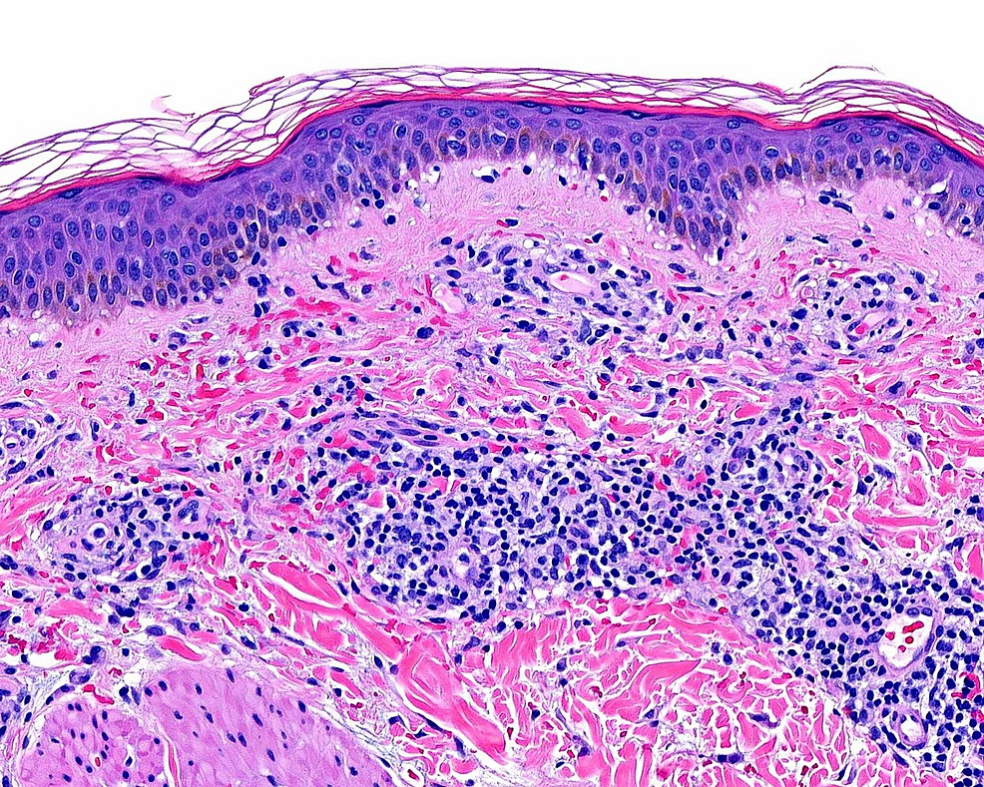
Schamberg disease histopathology. Superficial perivascular infiltrate of lymphocytes with numerous extravasated erythrocytes, some along the dermoepidermal junction (H&E: 200× magnification). H&E, hematoxylin and eosin

A 19-year-old woman presented with a two-week history of a progressive, asymptomatic rash on the bilateral legs, arms, and abdomen. Physical examination consisted of non-blanching erythematous and brown papules and macules that were most apparent on the lower extremities (Figure 1). As leukocytoclastic vasculitis was in the clinical differential diagnosis, two punch biopsies were performed for histopathology and direct immunofluorescence. The patient was initiated on a prednisone taper. Direct immunofluorescence was negative for IgG, IgA, IgM, and C3. Histopathology evaluation revealed superficial perivascular infiltration of lymphocytes with extravasated erythrocytes, consistent with Schamberg disease (Figure 2).

**Figure 3:**
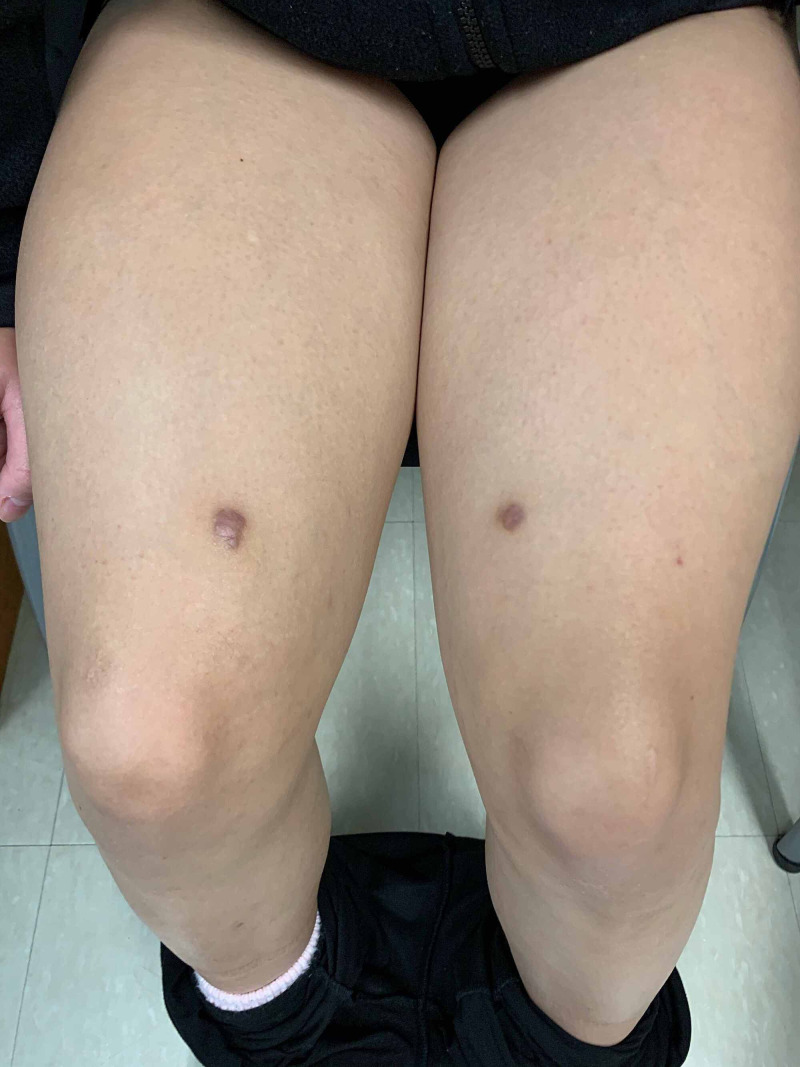
Schamberg disease. Four months post rutoside and ascorbic acid treatment. Two hyperpigmented scars at the sites of punch biopsies.

At the 1.5-month follow-up, the patient endorsed some improvement of her lesions with the 10-day taper of prednisone. However, her lesions quickly recurred soon after. At that time, she was initiated on oral ascorbic acid 500 mg twice daily and oral rutoside 50 mg twice daily. After three months of treatment, there was significant improvement in the patient’s lesions with complete lesion clearance at four months (Figure 3).

## Discussion

Few studies have tested the efficacy of rutoside and ascorbic acid in the treatment of PPD. A 1999 pilot study demonstrated that among three patients with variable duration of disease courses, full remission was achieved within four weeks of treatment, and remission was maintained at three months post-treatment [4]. Another study conducted between 2004 and 2011 found that 71.4% of its 35 participants treated with rutoside and ascorbic acid achieved full clearance of all lesions with a mean response rate of 7.9 weeks and mean treatment duration of 8.2 months [5].

The effectiveness of these treatments is related to both the hypothesized pathogenesis of PPD and the effect these agents have on cutaneous vasculature. PPD is thought to be due in part to the fragility of cutaneous blood vessels [6]. A contributing factor in the damage of vasculature is the generation of reactive oxygen species (ROS). ROS are known to damage blood vessels by inducing oxidative stress which, in turn, damages DNA and induces inflammation. ROS have been implicated in other vascular diseases such as atherosclerosis, diabetes mellitus, and hypertension [7,8]. Antioxidants, such as ascorbic acid and rutoside (a flavonoid) have the ability to scavenge ROS [9,10]; this helps to protect vasculature from ROS by preventing them from inflicting damage. While this mechanism of scavenging has been shown to be effective and even curative in the treatment of PPD, it currently is not the standard of care. The current standard of care (e.g., topical steroids and trigger avoidance) targets symptom control rather than an overall cure.

Current standard treatment for Schamberg disease includes topical steroids for those with pruritus, avoiding or discontinuing possible triggers (e.g., acetaminophen, aspirin, alcohol ingestion), and the use of compression stockings for vessel support [3]. However, these treatments may not provide a benefit to all patients. Compression stockings are temporary and may not provide long-term benefit after removal.

## Conclusions

The lack of any known severe side effects of rutoside and ascorbic acid for the treatment of PPD further supports its transition to standard of care. Studies suggest a linear correlation between time to response to treatment and disease duration. Our patient achieved significant improvement after approximately 12 weeks of treatment. Continued research, including randomized, controlled, clinical trials, would be justified to further demonstrate the efficacy of this treatment regimen. Exploration of different doses can help optimize treatment for the fastest and most effective treatment of PPD.
